# Relay Protection Device Reliability Assessment Through Radiation, Fault Injection and Fault Tree Analysis

**DOI:** 10.3390/mi16010069

**Published:** 2025-01-08

**Authors:** Hualiang Zhou, Hao Yu, Zhiyang Zou, Zhantao Su, Zheng Xu, Weitao Yang, Chaohui He

**Affiliations:** 1 State Grid Electric Power Research Institute, NARI Group Corporation, Nanjing 211106, China; 2 NARI Technology Co., Ltd., Nanjing 211106, China; 3 School of Nuclear Science and Technology, Xi’an Jiaotong University, Xi’an 710071, China

**Keywords:** relay protection device, α-particle, fault injection, single event upset, fault tree analysis

## Abstract

Relay protection devices must operate continuously throughout the year without anomalies. With the integration of advanced technology and process chips in secondary equipment, new risks need to be addressed to ensure the reliability of these relay protection devices. One such risk is the impact of α-particles inducing single event effects (SEEs) on the secondary equipment. To date, there has been limited assessment of the effects of α-particles on relay protection devices from a system perspective. This study evaluates the impact of SEE on relay protection devices through a Monte Carlo simulation, which is verified by α-particle radiation, fault injection, and fault tree analysis. It discusses the influence of SEEs with and without hardening measures in place. Additionally, this study examines the soft error probability when the target processor runs both general workloads and specific application workloads. The current research proposes a low-cost and effective reliability assessment method for secondary equipment considering single event effects. The findings provide new insights for the enhancement of future electric power grid systems.

## 1. Introduction

Secondary equipment in electric power grids plays a crucial role in monitoring, protection, control, and communication functions, ensuring the safe and efficient operation of primary equipment [1,2,3]. In recent years, there has been increasing demand for higher computing power and lower power consumption from power systems [4,5,6,7]. To meet this demand, related devices have increasingly embraced advanced semiconductor manufacturing processes and modular design [8,9].

The reliability of the secondary equipment, however, currently faces new challenges. Microprocessor-based relays, for instance, can be affected by single event effects (SEEs) caused by atmospheric neutrons or α-particles generated from radioactive impurities in packages of the chip, such as U and Th [10,11]. During decay, these impurities emit high-energy α-particles that, when striking the sensitive nodes of the chip, deposit energy and generate electron–hole pairs. This process can lead to SEEs.

In 2018 and 2020, SEE incidents were recorded in Chinese relay protection devices, highlighting the urgent need to assess the impact of SEEs on these devices. While some previous studies have examined the SEE influence induced by neutrons in the atmospheric environment [12,13,14], our current research emphasizes the assessment of the SEE impact from α-particles generated within the packaging on the reliability of Chinese relay protection devices, especially from the system and application perspectives. These are extremely lacking in existing relevant research.

Owing to the uncertainties associated with irradiation tests, including variations in irradiation duration and costs, the primary focus of the current study is on using irradiation experiments to verify whether α-particles can induce SEEs in a similar manufacturing technology with a core processor of targeted secondary equipment. Building on irradiation experiments, the following research emphasizes the outcomes of Monte Carlo simulations and software fault injection. Compared with irradiation tests alone, Monte Carlo simulation combined with software fault injection provides more detailed information that might be difficult to obtain through irradiation tests [15,16,17,18]. Furthermore, software fault injection can trigger a broader range of events inside the relay protection process or system, including failures to act, false operations, and others. In this study, an SEE assessment framework was developed for relay protection devices. This incorporates Monte Carlo simulation of α-particles generated in the chip package, software fault injection of α-particle-induced single event upsets (SEUs), as well as system assessment from a system perspective, combining the specific applications in the secondary equipment. This can provide new insights into the reliability assessment of secondary equipment in power systems.

## 2. Alpha Particle Irradiation

SEU irradiation experiments were conducted using ^241^Am α-particle sources targeting a system-on-chip (SoC) that employed the same technology process as the relay protection device. Before irradiation, the SoC was de-capped and the ^241^Am α-particle sources covered the top of the bare chip [19]. The particle flux of the ^241^Am α-particle source was 3759.8 cm^−2^·s^−1^. The emitted α-particles had a Linear Energy Transfer (LET) value of approximately 0.576 MeV·cm^2^·mg^−1^, with a range of 27.93 μm in silicon. Irradiation was performed on the 64 kB memory of the SoC, and the duration was 324 min with a final cumulative fluence of 7.31 × 10^7^ cm^−2^.

Different types of SEU were observed during the irradiation. Specifically, single-bit upset (SBU), dual-cell upset (DCU), triple-cell upset (TCU), and single event functional interruption (SEFI) events were detected. Table 1 lists the details of the events that were investigated. The corresponding SEU cross section was 2.48 × 10^−11^ cm^2^/bit.

From Table 1, it can be confirmed that α-particles emitted from the packaging can induce SEEs in the same process chips utilized in the secondary equipment. Nevertheless, to date, a comprehensive assessment of α-particle-induced SEEs in relay protection devices is lacking. For example, how the SEE in a memory block impacts the specific applications of the secondary equipment, how to quantitatively evaluate its propagation influence, and other issues should be investigated.

Compared with the irradiation tests to verify the SEE occurrences inner the relay protection device, the propagation mechanisms of system-level SEEs across different modules in the device can be investigated through simulation and fault injection techniques. In particular, it is combined with probability safe analysis solutions, such as fault tree analysis.

## 3. Monte Carlo Simulations

The Geant4 (Version@Geant4.10.5) toolkit can be applied to evaluate SEEs in the target relay protection device [20,21,22]. In the current research, it follows the same model that was adopted in [12]. The simulation model is based on the actual package structure of the target device. Based on the simulation model, during the α-particles simulation, 1 K and 1 M sensitive volumes were conducted for comparison. Each sensitive volume measured 160 nm × 160 nm × 160 nm in size in the simulation model, and the critical charge was 3820 eV. The sensitive volume and the critical charge are not chosen arbitrarily, the reliability of these parameters also needs to be confirmed by irradiation experiments and Geant4 simulations. Specifically, they are verified in [12] for the target device and iterated from the same technology SRAM irradiation tests results in [17,18,19,20,21] and others. Thus, they can be applied in this project.

Although the ^241^Am is one of the most widely available α sources, the ^232^Th is an important impurity in the packaging materials which can emit a majority of 4.013 MeVα-particles. This work aims to mimic α-particles inducing SEEs from package impurities. Hence, the energy of the α-particles was set to 4.013 MeV in simulation, and the corresponding LET was 0.70 MeV·cm^2^·mg^−1^. The physic process which was utilized contained ionization, decay, Coulomb scattering, etc. A total of 10^8^ impinging particles were generated at various angles.

**Figure 1:**
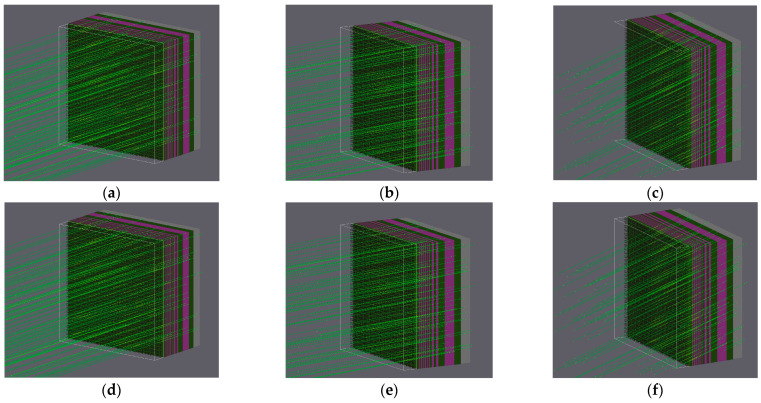
The α-particles impinging from different deflection angles: (**a**) from 0°; (**b**) from 15°; (**c**) from 30°; (**d**) from 45°; (**e**) from 60°; (**f**) from 75°.

As α-particles can be emitted from any direction in chip packaging, different incoming angles (angular deviation from the normal line of the chip surface), as shown in Figure 1, were used in the simulation to comprehensively evaluate the impact of particle incidence angles on SEUs.

At last, various SEU events were detected. Specifically, up to 18 simultaneously upset bits were observed in the 1 Kbits’ simulation, and 22 simultaneously flipped bits were investigated in the 1 Mbits’ simulation. The details of the recorded SEUs are listed in Table 2 and Table 3.

**Figure 2:**
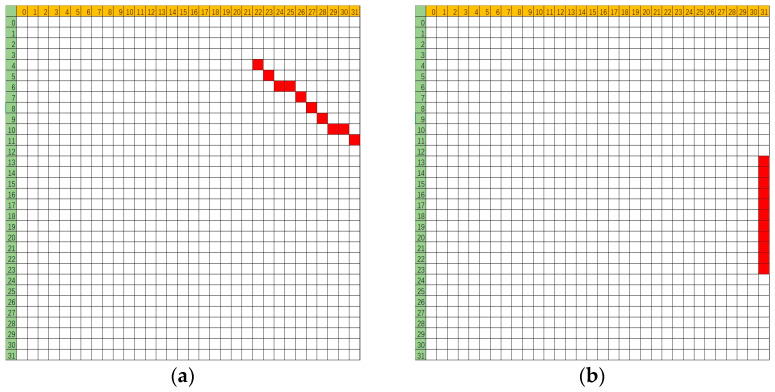
The distribution of different MCUs at the same incidence of particle striking, in where vertical is the bit offset and horizontal is the word offset: (**a**) 10-bit upset, (**b**) 11-bit upset.

Figure 2 illustrates the flipping bit distribution schematic diagrams of several multiple-cell upsets (MCUs), which may be distributed over multiple words and have different forms of distribution.

## 4. Software Fault Injection

For the SoC chip deployed in secondary equipment, the actual workload may not fully utilize the entire memory capacity, and not all SEUs occurring in the workload will lead to malfunction. It is necessary to combine software fault injection to evaluate and analyze the fault manifestation probability within the actual workload in the relay protection device.

### 4.1. Actual Failure and Fault Injection

The evaluation was carried out using the above-mentioned framework for assessing the SEE in relay protection devices, with fault injection performed on memory cells based on fault injection and Monte Carlo simulation results. Because of the high proportion of SBUs caused by α-particles within the packaging, and because SBUs can be eliminated with the use of error-correcting codes (ECC), this phase focuses solely on the soft errors resulting from MCUs in the equipment. It is worth noting that the SEU cross section of the device under α-particle striking, derived from Monte Carlo simulations, does not directly represent the failure rate during the actual operation of the secondary equipment, as not all soft errors necessarily led to functional failures that may have triggered anomalies such as false tripping or refusal to trip. Therefore, the fault manifestation probability, which is the likelihood that soft errors translate into perceivable failures, was obtained using software fault injection methods. This probability depends on multiple factors, including, but not limited to:

Error masking: In certain cases, even though soft errors occur because of logical relationships or how data are used, these errors may have no discernible impact on the operational process or program output, or the erroneous data may be overwritten by new data before causing an exception.

Error tolerance and recovery technologies: System design may include specific redundancy or fault-tolerance techniques, such as ECC or other types of error correction technology, which can reduce the probability of soft errors manifesting as failures.

Position of the soft error: The significance of the data or execution path affected by the error also influences its manifestation; for example, the soft error that occurs in hardware regions outside the active workload will not cause exceptions.

### 4.2. Fault Injection in General Test Programs

The Fast Fourier Transform (FFT) is widely used in secondary equipment for applications such as harmonic detection, fault detection and location, system stability analysis, and load monitoring and analysis [23]. Consequently, a general test program based on FFT was developed in C language. The main functionalities of the program are as follows:

➢ The authentication section simulates user input and verifies credentials;
➢ The I/O reading section acquires time-domain signals of voltage and current;
➢ The data analysis section checks instantaneous values and performs FFT;
➢ The power calculation section computes active power, root mean square values, apparent power, and power factor based on the available data.

The general test program operates with a cycle time of approximately 1000 ms.

In the implementation of ECC, the Single Error Correct–Double Error Detect (SEC-DED) code was used [24], where each 64-bit data segment underwent parity calculation to produce the first 7 bits of checksum, and the 8^th^ bit was a parity bit that checked all other data bits. The data and checksum bits (64-bit + 8-bit) were stored separately in different members of a structure. During fault injection, logic that may induce errors in the checksum bits was added. In the ECC checking process, syndromes were calculated for error detection and correction, as shown in Table 4. It is important to note that, in cases where an odd number of bit errors greater than or equal to three occurs, it may not be possible to identify or calculate the error location, though the number of errors in the final data will not exceed the number of original erroneous bits, which might occur in fault injection.

During the fault injection testing process, the pattern of bit flips was based on the coordinates of DCUs and MCUs in the memory cell array obtained from Geant4 simulation results. For each bit to be flipped in DCU or MCU, the corresponding byte and bit positions were calculated, and each designated bit was flipped using XOR operations. The ratios of dual-bit and multi-bit flips used in the tests reflect the outcomes of various particle incidence angles derived from Geant4 simulations.

**Figure 3:**
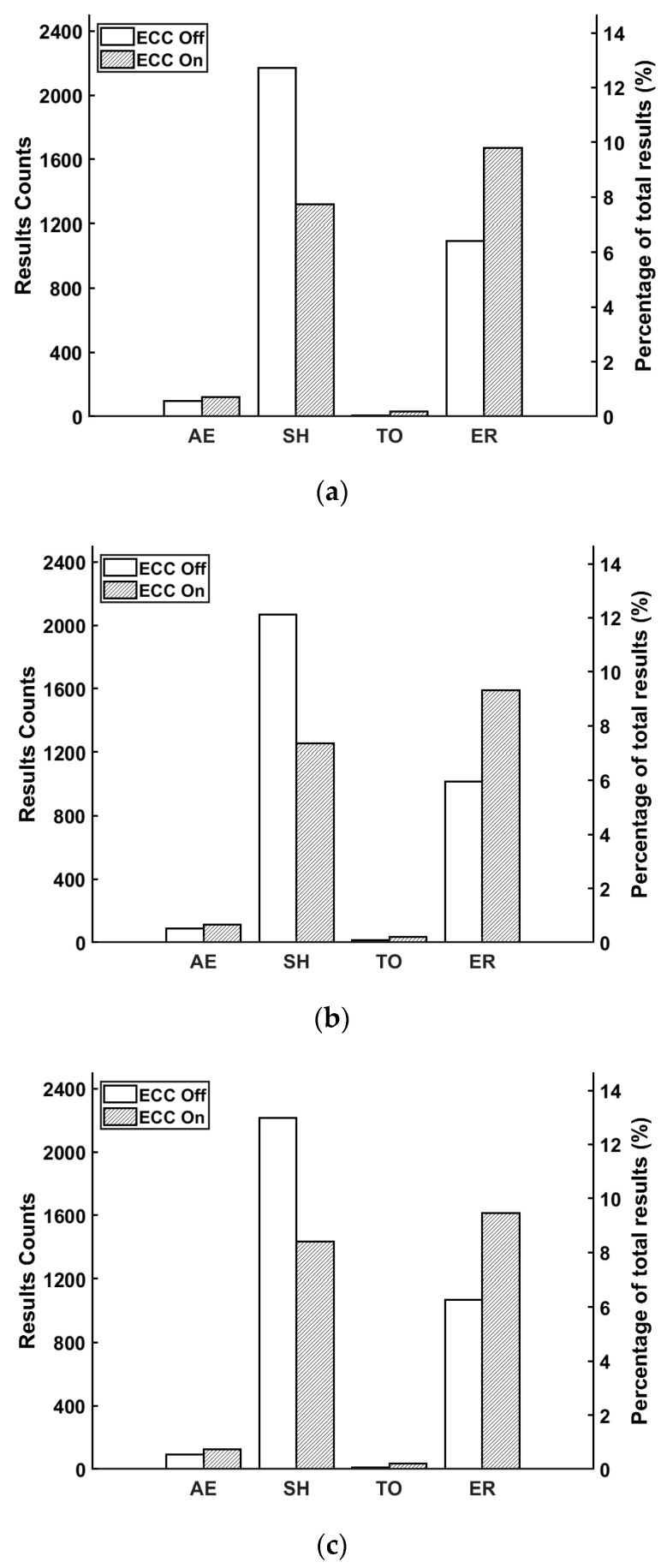
Different deflection angles of impinging α-particles were set in the fault injection testing; four abnormal results were detected: (**a**) from 30°; (**b**) from 60°; (**c**) from 90°.

The fault injection process involved changing the deflection angles of impinging particles and whether ECC was activated. A total of 16,385 fault injections were conducted, with the proportion of faults varying according to different deflection angles as follows: At a deflection angle of 30°, DCUs accounted for 91.93% and MCUs for 9.07%; at 60°, DCUs constituted 99.96% and MCUs 0.04%; and at 90°, DCUs represented 86.09% and MCUs 13.91%. As shown in Figure 3, five results were detected during the fault injection in general test programs, including abnormal exit (AE), system halt (SH), time out (TO), error result (ER), and normal [12].

➢ Abnormal exit (AE): Program exit code experiences an abnormality.
➢ System halt (SH): Program execution is halted.
➢ Time out (TO): Program execution is out of the expected duration.
➢ Error result (ER): The execution results are different from the expected.
➢ Normal: The injected faults have no visible influence on the tested program’s execution.

**Figure 4:**
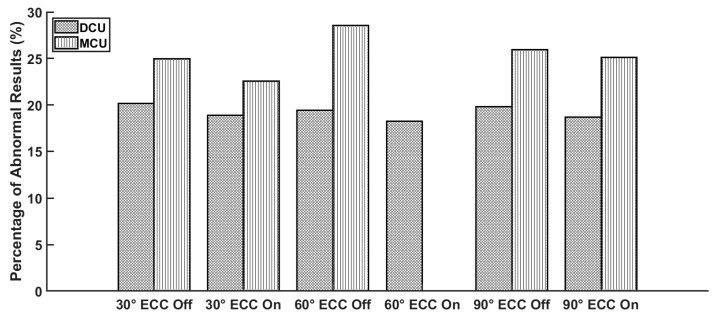
The number of abnormal results caused by DCUs and MCUs varied under different conditions.

Among them, the first four soft errors are abnormal for the secondary equipment. Figure 4 shows the abnormal results caused by DCUs and MCUs in different cases.

As depicted in Figure 3, the occurrence probability of SH and ER was significantly higher than that of AE and TO. For both DCUs and MCUs, the use of ECC effectively reduced the occurrence of SH, potentially converting SH into one of the other three abnormal outcomes (mainly ER). Figure 4 illustrates that MCUs were more likely to cause abnormal results compared to DCUs, and in practice, ECC did not significantly reduce the total number of errors.

### 4.3. Fault Injection in Actual Workloads

**Figure 5:**
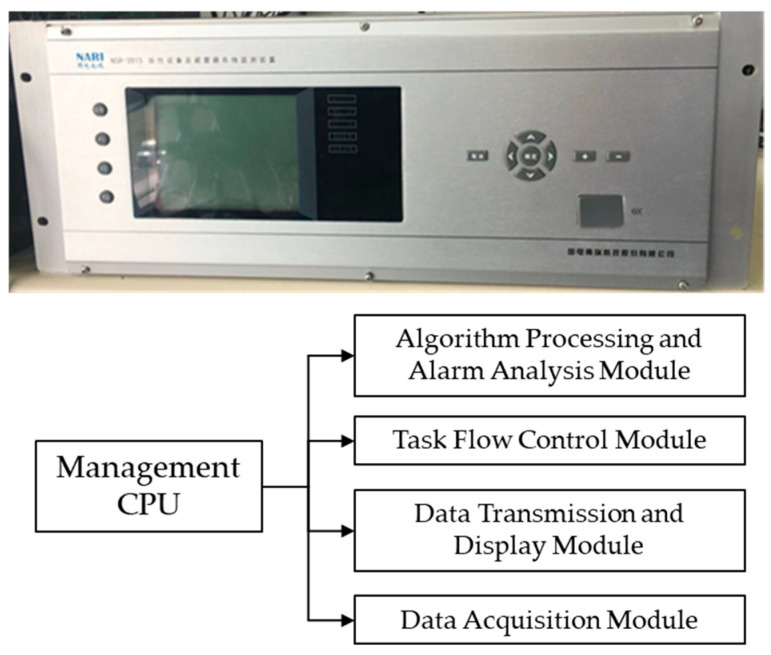
Different modules share the same program space in the device. (**Top**) is the photo of the device, and the (**bottom**) is the specific workloads.

A test platform was constructed using the actual operational software of secondary equipment, featuring an embedded system design identical to the real product. The testing system consisted of a real-time CPU core (bare metal) and a management CPU core (ARM side). The real-time CPU core, operating without an operating system, handled functions such as logic, alarms, and control, which require high real-time performance. On the other hand, the ARM side, based on an embedded Linux Operating System, was designed to manage, communicate, and display the functions of the device. The modules shown in Figure 5 represent the core function modules running on the management CPU core. They include the algorithm-processing and alarm analysis module, task flow control module, data transmission and display module, and data acquisition module.

Combining the specific characters of the test platform and the workload, the types of results have changed from five to three categories: System Halt (SH), Error Result (ER), and Normal. Table 5 displays the results of fault injection in core function modules.

Using the same test platform, fault injection tests were conducted on the bare-metal program. It employed variable swapping to introduce errors into the operation of the real-time CPU core. The operation states were observed, and only SH appeared in the test with a count of 295, while the total test count was 504.

**Figure 6:**
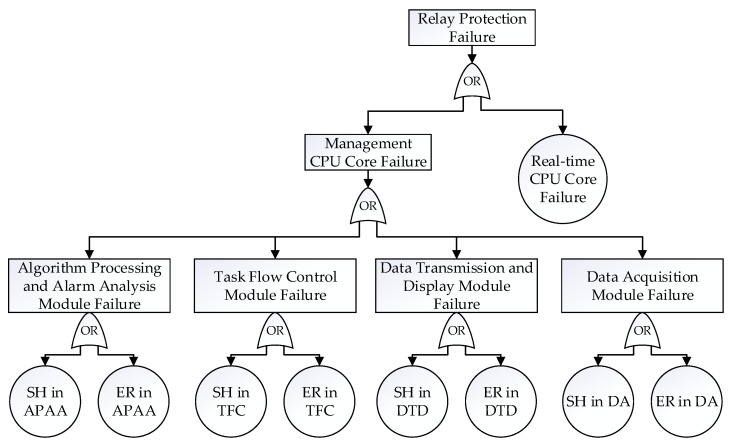
The fault tree of a secondary equipment system comprising two main components, including a management CPU core and a real-time CPU core.

To quantitatively analyze the system’s reliability, the fault tree was built. Based on the probabilities of various abnormal results, a fault tree analysis model, as illustrated in Figure 6, was constructed to analyze the failures throughout the entire secondary equipment system [25]. This fault tree model helped to identify potential vulnerabilities and assess the overall reliability of the system.

The impact of each module’s soft error on the system’s reliability based on the fault injection results is provided, and the sensitive modules were observed directly in this fault tree. The entire probability of system failure was approximately 33%, considering the multi-module’s failure from the management CPU core and the single failure from the real-time CPU core. Each module contributed differently to system failures in the management CPU core. In specific, the task flow control module had the greatest impact on SH (7.61%), and the data acquisition module experienced a significant probability of causing ER (2.46%). The failure of real-time CPU core directly caused SH (11.69%), and although its impact probability was lower than the total probability of management CPU core failure (21.36%), its influence as a single source of failure was significant.

## 5. Discussion

From Table 2 and Table 3, it can be observed that α-particles deflected by 15° induced the highest number of bit flips owing to SEEs. For a 1 K sensitive volume array, the SEE cross section under a 15° deflection of α-particles was 2.16 × 10^−10^ cm^2^/bit, while for a 1 M sensitive volume array, it was 2.67 × 10^−10^ cm^2^/bit. These results indicate that, for α-particles, the simulation outcomes for 1 K and 1 M sensitive volume arrays were similar, primarily because α-particle-induced SEEs resulted from direct ionization and were mainly related to the Linear Energy Transfer (LET), showing negligible dependency on capacity. Additionally, from the statistics of SBUs, DCUs, and MCUs caused by α-particle incidence, it is evident that SBUs played a significant role across different incidence scenarios.

For the α-particle irradiation test in Section 2, the SEU cross section was 2.48 × 10^−11^ cm^2^/bit. In the simulation, the cross section corresponding to the highest number of bit flips was 2.16 × 10^−10^ cm^2^/bit. The LET in the irradiation test was 0.576 MeV·cm^2^·mg^−1^, while in the simulation, it was 0.70 MeV·cm^2^·mg^−1^. The slightly higher LET in the simulation resulted in a slightly higher SEU cross section. This confirms that the constructed simulation was reasonable, and the subsequent fault injection and analysis were also valid.

Additionally, the failure rate could be estimated based on the simulation results. Since the 28 nm CMOS SRAM was packaged with an α particle emission rate of 0.001 α/cm^2^·h, deploying approximately 10^5^ CPU board chips with a memory capacity of 1 M bits using the same technology could result in 270 SEUs per year. Each SEU had a probability of approximately 99.989% as an SBU, approximately 0.01% as a DCU, and less than 0.01% as an MCU.

Software fault injection revealed how the test load responded to the DCUs and MCUs induced by α-particles. By evaluating ECC without considering SBUs, it was found that ECC did not significantly reduce the abnormal results caused by DCUs and MCUs in the test load. However, ECC was able to mitigate the severity of these events to some extent. Based on the fault injection results of the actual workload, a fault tree was established. Analysis of this fault tree indicates that, in the event of a DCU or MCU, the real-time CPU core in the secondary equipment is most likely to cause a failure to act because of an SH. In contrast, the data acquisition module is most likely to cause a false operation owing to the ER.

In summary, when evaluating the reliability of the relevant secondary equipment systems, especially when there are no guidelines or standards for this area, the recommended implementation measures are as follows: Firstly, it calculates the SEU cross section for some bits’ memory cell arrays to quantitatively assess its sensitivity to SEUs. Secondly, it conducts software fault injection testing to determine the failure rates of different software architectures or versions under conditions of SEUs while considering the occupancy rates of various software loads to evaluate overall software robustness. Thirdly, it examines the effectiveness of various mitigation techniques, such as ECC, periodic refreshing, and triple voting systems, in alleviating the adverse effects of SEUs, and assesses the performance of these techniques under actual workload conditions. Last but not least, it builds a fault tree to quantitatively assess the influence propagation by SEE in memory cells.

Furthermore, it should be clarified that the proposed solution in this paper is not limited to relay protection devices; it is also applicable to a wide range of microprocessor-based devices, including SoCs and other integrated systems. While the paper primarily focuses on relay protection devices, it takes the following two points into account: First, relay protection systems are high-reliability systems that are increasingly susceptible to SEE threats. Second, the paper aims to remind researchers to consider the effects of SEE on terrestrial environments, such as relay protection devices, in addition to aerospace electronic systems.

## 6. Conclusions

SEEs induced by α-particles on a relay protection device chip in secondary equipment were assessed. Through Monte Carlo simulations, the results of SEUs caused by α-particles striking various deflection angles were obtained. The simulation results indicate that α-particles are more likely to trigger SEUs when they are incident at a 15° deflection angle. Based on the Monte Carlo simulation data for SEUs, fault injection was performed on a general test program and real workload conditions, cataloging various anomalous results, such as abnormal exit, system halt, time out, and error results. The results suggest that, to enhance the mitigation effectiveness against soft errors induced by DCU or MCU, the chip would require more robust ECC or other hardening measures. Fault tree analysis identified the modules within the core CPU of the secondary equipment that were the most susceptible to different errors during operation. The Monte Carlo simulation method is universally applicable, and the software fault injection framework can be ported to similar systems, providing reliability assessments for SEUs in a variety of secondary equipment, not limited to relay protection devices.

## Figures and Tables

**Table 1:** The observed single-event effects in alpha irradiation tests.

SBU	DCU	TCU	SEFI
951	55	1	13

**Table 2:** Numbers of different types of SEUs occurring at different deviation angles in the 1 Kbits’ simulation.

SEU	0°	15°	30°	45°	60°	75°
1	26,327,357	26,372,693	25,367,079	20,683,590	1,889,455	625
2	1504	1649	3341	57,809	214,264	10
3	171	186	218	301	44	-
4	51	38	43	59	9	-
5	11	13	19	18	1	-
6	3	7	8	11	1	-
7	2	4	1	5	-	-
8	1	1	1	1	-	-
9	1	3	-	5	-	-
10	-	1	1	3	-	-
11	2	2	-	-	1	-
12	-	1	-	-	-	-
13	1	-	-	1	-	-
14	-	-	1	1	-	-
15	-	-	1	1	-	-
18	-	1	-	-	-	-

**Table 3:** Numbers of different types of SEUs occurring at different deviation angles in the 1 Mbits’ simulation.

SEU	0°	15°	30°	45°	60°	75°
1	26,008,579	26,068,611	25,244,039	20,791,694	2,153,906	520
2	2093	2192	4039	59,345	267,078	9
3	296	299	311	399	82	1
4	91	84	69	81	14	-
5	32	25	33	28	5	-
6	14	13	10	15	4	-
7	11	13	10	3	-	-
8	1	1	4	6	-	-
9	4	2	3	4	-	-
10	1	3	3	-	-	-
11	1	1	3	2	-	-
12	-	-	-	2	-	-
13	-	-	1	-	-	-
14	-	-	1	1	-	-
17	-	-	1	-	-	-
18	-	-	-	2	-	-
22	-	1	-	-	-	-

**Table 4:** Check results indicated by different syndromes.

Syndrome Phenomenon	Check Result
No error indicated in syndrome [6:0]	Data are correct.
No error indicated in syndrome [7]	
Error indicated in syndrome [6:0]	DCU is detected.
No error indicated in syndrome [7]	
No error indicated in syndrome [6:0]	SBU is detected and corrected.
Error indicated in syndrome [7]	
Error indicated in syndrome [6:0]	SBU is detected and corrected.
Error indicated in syndrome [7]	

**Table 5:** Results of fault injection in different modules in management CPU core.

Module Name	Count of SH	Count of ER	Count of Normal
Algorithm Processing and Alarm Analysis (APAA)	110	9	357
Task Flow Control (TFC)	192	12	948
Data Transmission and Display (DTD)	62	4	70
Data Acquisition (DA)	88	62	106

## Data Availability

The data used to support the findings of this study are available from the corresponding author upon request.
